# Impact of Using Population-Specific Cut-Points, Self-Reported Health, and Socio-Economic Parameters to Predict Sarcopenia: A Cross-Sectional Study in Community-Dwelling Kosovans Aged 60 Years and Older

**DOI:** 10.3390/jcm11195579

**Published:** 2022-09-22

**Authors:** Arben Boshnjaku, Abedin Bahtiri, Kaltrina Feka, Ermira Krasniqi, Harald Tschan, Barbara Wessner

**Affiliations:** 1 Centre for Sport Science and University Sports, University of Vienna, 1150 Vienna, Austria; 2 Vienna Doctoral School of Pharmaceutical, Nutritional and Sport Sciences (PhaNuSpo), University of Vienna, 1090 Vienna, Austria; 3 Department of Physiotherapy, Faculty of Medicine, University “Fehmi Agani” in Gjakova, 50000 Gjakova, Kosovo; 4 Department of Sport and Movement Science, University for Business and Technology, 10000 Pristina, Kosovo; 5 Department of Psychology, Educational Science and Human Movement, University of Palermo, 90128 Palermo, Italy; 6 Sport and Exercise Science Research Unit, University of Palermo, 90144 Palermo, Italy; 7 Research Platform Active Ageing, University of Vienna, 1090 Vienna, Austria

**Keywords:** sarcopenia diagnostics, developing country, older adults, EWGSOP, population-specific cut points

## Abstract

The age-related decline of muscle strength, mass, and physical performance (sarcopenia) has been raising concerns among the scientific and healthcare communities. This decline may differ between populations, age groups, and sexes. Therefore, we aimed to explore sarcopenia together with the impact of health and socio-economic parameters in mature Kosovans. A cross-sectional study was conducted on community-dwelling adults aged ≥ 60 years (*n* = 240, 47.1% female) from the Prishtina region. Sarcopenia was identified using the following criteria: (i) the European Working Group in Sarcopenia for Older People (EWGSOP1), (ii) the revised EWGSOP2 algorithms, and (iii) sex-specific cut-points derived from the Kosovan population. In males, pre-sarcopenia/probable sarcopenia was detected from the EWGSOP1, EWGSOP2 and Kosovan-specific criteria at values of 3.1%, 5.5%, and 28.3%; sarcopenia was detected at 1.6%, 5.5%, and 0.0%, and severe sarcopenia was detected at 4.7%, 2.4%, and 4.7%, respectively. Pre-sarcopenia was lower in females (0.9%, 5.3%, 16.8%), with no cases of sarcopenia or severe sarcopenia detected by either algorithm. Sarcopenic males were older, had a lower weight, BMI, skeletal muscle mass, performance score, nutritional status (*p* < 0.001), educational level (*p* = 0.035), and higher malnourishment risk (*p* = 0.005). It is notable that high overweight and obesity levels were also detected (93.8% of females, 77.1% of males). This study highlights the importance of using population-specific cut-points when diagnosing sarcopenia, as otherwise its occurrence may be underestimated, especially in obese persons. Age, body composition, physical performance, health, and socio-economic conditions can influence the occurrence of sarcopenia.

## 1. Introduction

Sarcopenia presents an issue that is rapidly gaining interest among the scientific community, and it is clinically used to describe the age-related changes in the skeletomuscular system and their impact on the health of older people [1]. In 2010, a practical clinical definition and consensus diagnostic criteria for age-related sarcopenia was developed by the European Working Group on Sarcopenia in Older People (EWGSOP1) [2]; it focused on lower muscle mass as the key characteristic accompanied by either lower muscle strength or worse physical performance (PP), or both (sarcopenia and severe sarcopenia, respectively). The revised 2019 version (EWGSOP2) [3] defined sarcopenia as a muscle disease (muscle failure) rooted in adverse muscle changes that occur across a lifetime, which was also associated with an increased likelihood of adverse outcomes, including falls, fractures, physical disability, and mortality. With the EWGSOP2 criteria, the attention was shifted towards the recommendation of using muscle strength as the primary parameter for sarcopenia diagnosis (probable sarcopenia). Low muscle quantity or quality would confirm the condition (confirmed sarcopenia), and the combined presence of low muscle strength, low muscle quantity/quality, and low physical performance characterizes the state of severe sarcopenia [3]. In addition to modifying the diagnostic algorithm, population-specific cut points were also proposed for use, namely to assess two standard deviations below the mean of a young comparison population [4].

Global estimates of sarcopenia prevalence vary from 10% to 27% depending on the definition used, ethnicity, age, and sex [5]. Sex emerges as a particularly interesting sarcopenia influence factor because of the age-related hormonal effects on muscle phenotypes, notably through estrogens and estradiol in particular [6,7]. However, ageing is a process that, aside from the progressive decline of muscle quality and quantity, is often characterized with a sex-specific visceral fat increase as well. Visceral fat itself has been shown to be related and even induce inflammation [8], which may subsequently contribute to the development of sarcopenia [9]. Additionally, it has been shown that a population’s general health status as well as their educational and socio-economic status might affect the prevalence levels [10,11]. To date, the majority of studies have been predominantly based on Caucasians from high-income countries [12], but data from low- and middle-income countries involving a Caucasian population within Europe are scarce [13,14]. Kosovo declared independence in 2008 and has been recognized by more than 100 United Nations members as well as 23 out of 28 members of the European Union (EU). Despite its development to an upper-middle-income country and experiencing a solid economic growth over the last decade, life expectancy is still in the lower range compared with the EU (70.3 years for males and 74.8 years for females) [15]. Sarcopenia has been associated with an increased risk of all-cause mortality [16], but no study so far has determined neither its occurrence nor the more robust diagnostic criteria and cut-points for the Kosovan population. Therefore, this study aimed to determine sarcopenia levels as defined by different diagnostic criteria (EWGSOP1, EWGSOP2, and population-specific cut points) in older Kosovan adults and evaluated the impact of socio-economic, health-related, and lifestyle factors on sarcopenia status.

## 2. Materials and Methods

### 2.1. Study Design and Sample

This cross-sectional study examined data from community-dwelling males and females aged over 60 years living in the region of Prishtina, the largest region in Kosovo. This region is home to 477,312 people (27.4% of the total population of Kosovo), and 6.7% of the total Kosovan population are over 60 years old [17]. An a priori sample size estimation has been performed by using the following equation: n = (z^2^) × P × (1 − P)/d^2^, where n = sample size, z = z statistic for the level of confidence (1.96), P = expected prevalence, and d = allowable error. Hence, for an expected prevalence between 10% and 90% (P = 0.1 to 0.9), a d of ±5% has been suggested as a reasonable choice [18]. Therefore, a sample size of *n* = 144 was considered necessary to detect a 10% prevalence. However, we enabled participation for all interested persons that fulfilled the inclusion criteria. In total, 240 adults (113 females and 127 males) aged ≥60 years participated, representing about 0.8% of the eligible sample. Participants were recruited through announcements (written and verbal) in the Kosovo Pensioners’ Association branches in Prishtina and Fushë Kosova (neighboring municipality within the same region), Prishtina Nursing Homes, and national TV broadcasting. This recruitment strategy intended to notify every eligible participant for the possibility to take part in the study and thus avoid a potential healthy subject bias through the most approachable means: associations (of which aside from the formal offices also includes a restaurant, coffee shop, and two board game rooms), one of the most frequented places during the daytime; nursing homes, the places where some of the target community lives; and TV programs, which could explain the study and approach towards the interested participants within their homes. Inclusion criteria were set in accordance with the study’s specifications and measurement techniques and participants were assessed at the entrance by two healthcare experts; the assessment involved determining the sex, age, living region, and lack of any underlying condition or disease that could prohibit the participant from performing measurements (e.g., any cardiac implantable electronic devices).

### 2.2. Data Collection and Anthropometric Measurements

Data collection was organized at the Sports Medicine Laboratory at Universi College in Prishtina. All measurements were uniformly performed with respect to the testing order by the same research team during the entire data collection period. Anthropometric measurements were performed in accordance with the International Standards for Anthropometric Assessment in the morning after an overnight fast, starting with height, weight, and body composition [19]; subjects wore light indoor clothing and were barefoot. Heights were measured with a precision of 5 mm using the stretch stature method with a portable stadiometer (DT05L, Kinlee, Zhongshan Jinli Electronic Weighing Equipment Co. Ltd., Zhongshan, China). Body compositions were measured using segmental multi-frequency bioelectrical impedance analyses (BIA), with operating frequencies of 1, 5, 50, 250, 500 kHz and 1 mHz using a device that also allowed for the assessment of body mass (Inbody 720, Biospace Co., Seoul, Korea). Body composition variables included the whole body skeletal muscle mass (SMM) and fat mass. Appendicular skeletal muscle mass (ASMM) was assessed as the sum of the lean mass of both arms and legs. In order to predict the dual-energy X-ray absorptiometry (DXA) estimation of muscle mass when measuring by the BIA method, a multivariate regression model was used, as previously evaluated [20,21]. Body mass index (BMI), skeletal muscle index (SMI), and appendicular skeletal muscle index (ASMI) were determined as the body mass, SMM, or ASMM divided by height squared (all expressed as kg/m^2^) [22].

### 2.3. Physical Performance and Strength Measurements

After the anthropometric assessments, a light standardized meal was provided 30 min before starting the strength and physical performance measurements. The isometric handgrip strength was measured by using handgrip dynamometry (JAMAR, Patterson Medical, Saint Paul, MN, USA). Each participant was asked to perform two trials with 1 min of rest in between the trials. The adaptable dynamometer was squeezed for a maximal isometric contraction time of 4–5 s using the self-reported dominant hand. The better result of the two trials was considered for the analyses [23].

For assessing gait speed, participants were asked to walk in their usual gait speed on a path six meters long with an additional two meters for acceleration and deceleration (a total course length of 10 m). The time for the completion of the six meters was manually recorded by the tester using a stopwatch; the gait speed was expressed in m/s. The better result of the two trials was used [23].

For the timed up and go test (TUG), subjects were required to stand up from a chair without armrests upon the starting signal, walk three meters on a linear course, turn around, and return to the starting position, where they had to sit down again. The time taken to complete this task was measured in seconds using a stopwatch [24].

Lower body strength endurance was tested by the 30-seconds (30-s) chair stand test as previously described using an armless chair with a height of 46 cm and placed against a wall. Participants were instructed to stand up and sit down as often as possible within 30 s while having their arms crossed over their chest. Fully completed repetitions (including the last attempt if completing more than 50% of the task before the time expired) were counted by the tester who stayed next to the participant, holding a stopwatch and signaling the beginning and end of the time. Similarly, the 30-s arm curl test estimated the upper body strength endurance of the participants by counting the number of repetitions of elbow flexion with supination. The participants lifted a dumbbell (5 pounds for women and 8 pounds for men) while sitting upright on a chair upon receiving the starting and ending signal by the tester, who followed a similar protocol as the previous test (standing next to the participant and counting the repetitions, including the last repetition if the participant completed more than 50% of the task before the time expired). The task was performed for the self-reported dominant side [25].

For determining aerobic endurance, the 6-min walking test (6MWT) was used. Subjects were requested upon being signaled to walk as fast as possible for 6 min on a 30-meter shuttle track while being alone on the track. Testers strictly followed the process, counted the time, and signaled the ending. If necessary, the speed could be reduced or the person was allowed to rest if the selected speed was too high to be sustained, and the covered distance was reported in meters as the test outcome [26]. In four cases (1.7%), the use of a cane was necessary to perform the tasks that involved walking (gait speed, TUG, and 6MWT).

### 2.4. Assessment of Sarcopenia

Sarcopenia was diagnosed according to the EWGSOP1 and EWGSOP2 algorithms using the SMI/ASMI, handgrip strength, and gait speed tests, as indicated in the recommendations [2,3]. In order to compare the different modalities, we used either the suggested cut-points or population- and sex-specific cut-points for the Kosovan population. The latter were derived as suggested by Baumgartner et al. [4] through the subtraction of two standard deviations from the respective mean of a given parameter; we derived the cut-points from a young Kosovan sample [27]. Sex-specific thresholds were set to 5.74 kg/m^2^ (male) and 4.77 kg/m^2^ (female) for ASMI, 32.83 kg (male) and 19.64 kg (female) for handgrip strength, and 1.14 m/s (male) and 1.03 m/s (female) for gait speed.

### 2.5. Secondary Endpoints

The self-perceived health status and comorbidities were collected by two healthcare professionals. The nutritional status was assessed by the Mini Nutritional Assessment (MNA) questionnaire (long form), an 18 item tool comprising anthropometric measurements (BMI, weight loss, mid-arm, and calf circumferences) dietary intake (feeding autonomy, amount of food and fluids intake, number of meals consumed), general lifestyle assessment (living environment, medication consumption, mobility, pressure ulcers, presence of stress, depression, or dementia), and subjective self-assessment (self-perception of own health and nutritional level) [28]. Medication usage was assessed by the Brief Medication Questionnaire 1 [29]. The environment of living, education level, marital status, financial status and self-perceived poverty level, self-perceived health condition, comorbidities, smoking status, and alcohol consumption were collected and analyzed using the WHO STEPS instrument [30].

A physical performance score including the five performance tests (gait speed, TUG, 30-s arm curl test, 30-seconds chair stand test and 6MWT) was created using the weighted-sum method as has been previously described. Briefly, the test results of the individual tests were scaled to Z-scores and subjected to a principal component analysis in order to obtain the loading value of each test. The performance scores for each subject were calculated by multiplying the Z-scores with their associated loading value before summation [31].

### 2.6. Statistical Analysis

The means, standard deviations (continuous variables), and frequencies (categorical variables) were calculated to describe the general, anthropometric, and physical fitness variables. A chi-square test was used to compare the frequencies between groups. Differences between the continuous variables were defined by an independent *t*-test and one-way ANOVA test, the latter followed by Tukey post hoc analyses, which also suggested homogenous subgroups. The homogeneity of variances were checked by a Levene test. Effect sizes were calculated to estimate the magnitude of the effect. Cohen’s d was used when comparing males and females, whereas ϖ^2^ was applied together with a one-factorial ANOVA test. A multinomial logistic regression was used to ascertain the impact of various covariates on the sarcopenia status. “No sarcopenia” was set as the reference group, and financial condition and health condition were included as factors; age, BMI, body fat mass, SMM, PP, and MNA scores were included as covariates. As “no sarcopenia” is present in more than 10% of the cases, the odds ratio (OR) derived from the logistic regression might overestimate the risk ratio (when it is more than 1) or underestimates the risk ratio (when it is less than 1). Therefore, the relative risk (RR) was estimated using the formula provided by Zhang and Yu [32]: RR = OR/[(1 − P0) + (P0 × OR), where P0 indicates the incidence of no sarcopenia. The statistical significance was set at *p* < 0.05, and all data were analyzed using the statistical package SPSS 26 for Windows (SPSS Inc., Chicago, IL, USA).

## 3. Results

### 3.1. Participants’ Characteristics

**Figure 1:**
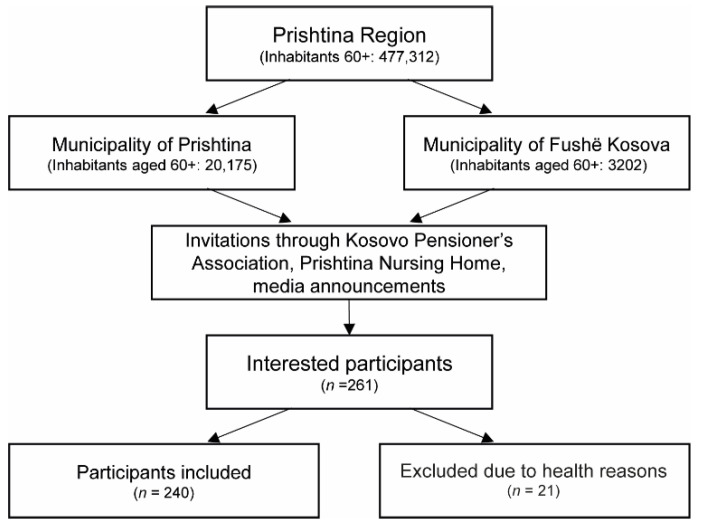
Selection of study participants.

Figure 1 shows the flowchart for study participants. From the total of 477,312 people aged 60 years and older within the region of Prishtina, 20,175 and 3202 subjects from the municipalities of Prishtina and Fushë Kosova, respectively, were eligible to participate. From those, 261 expressed their interest to participate, out of whom 240 persons fulfilled the inclusion criteria and took part in the study. Data from the first 61 of these participants were used to assess the reliability of the assessment parameters and calculate diagnostic cut-off points for sarcopenia [27].

The characteristics of the 240 subjects included in the study are summarized in Table 1. Males were observed to be older (t_238_ = 5.187, *p* < 0.001, d = 0.672) and taller (t_238_ = 15.438, *p* < 0.001, d = 2.001), with a higher SMM (t_218_._4_ = 12.801, *p* < 0.001, d = 1.732) and SMI (t_223_._9_ = 6.816, *p* < 0.001, d = 0.911) but a lower ASMI (t_238_ = −3.055, *p* < 0.001, d = −0.396). Females had a higher BMI (t_238_ = 7.733, *p* < 0.001, d = 1.003), whole body fat mass (t_238_ = 8.688, *p* < 0.001, d = 1.126), and fat percentage (t_232_._2_ = 14.741, *p* < 0.001, d = 1.935). No differences were found for body mass between the sexes (t_238_ = −0.670, *p* = 0.504, d = −0.087). Additionally, the females presented a lower nutritional score (from the MNA long form, t_238_ = −3.911, *p* < 0.001, d = −0.507) and consequently a higher prevalence of malnourishment and risk for malnourishment (*p* < 0.001) in comparison to the males. The female participants also reported a lower frequency of smoking (*p* = 0.001), lower level of education (*p* < 0.001), and worse financial condition (*p* = 0.022), but reported a higher medication intake compared with the males (*p* < 0.001). When observing the sex differences in physical performance, a significantly lower physical performance score was seen in females (t_238_ = −4.428, *p* < 0.001, d = −0.574), which was also the case for the individual performance tests (all *p* < 0.001, with Cohen’s d ranging from −1.794 to 0.391); the one exception was the 30-s arm curl test (t_238_ = −1.650, *p* = 0.100, d = −0.214).

### 3.2. Sarcopenia and Conceptual Stages in the Study Population

**Figure 2:**
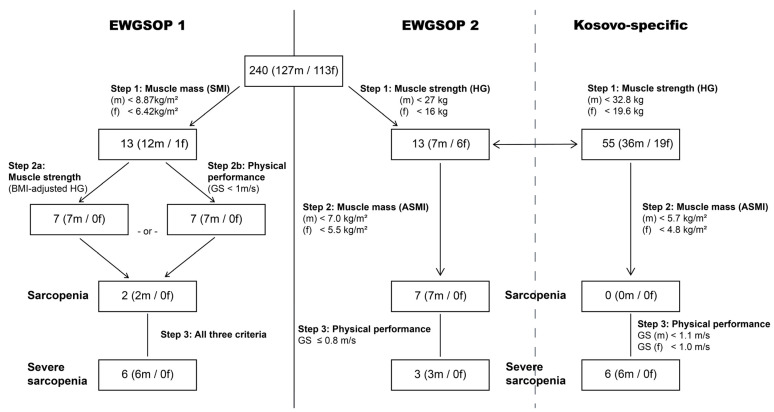
Sarcopenia case finding according to the EWGSOP1, EWGSOP2 and population-specific cut-points. EWGSOP1: pre-sarcopenia, low SMI; sarcopenia, low SMI and low HG or PP; severe sarcopenia, low SMI, low HG, and low PP. EWGSOP2 and Kosovo-specific: probable sarcopenia, low HG; sarcopenia, low HG and low ASMI; severe sarcopenia, low HG, low ASMI, and low PP. ASMI, appendicular skeletal muscle index; BMI, body mass index; GS, gait speed; HG, handgrip strength; SMI, skeletal muscle index; PP physical performance. Specific cut-points are given in the Figure. For the BMI-adjusted HG (EWGSOP1), the following values were used: males (m): ≤29 kg for BMI ≤ 24 kg/m^2^, ≤30 kg for BMI 24.1–28 kg/m^2^, ≤32 kg for BMI > 28 kg/m^2^; females (f): ≤17 kg for BMI ≤ 23 kg/m^2^, ≤17.3 kg for BMI 23.1–26 kg/m^2^, ≤18 kg for BMI 26.1–29 kg/m^2^, ≤21 kg for BMI > 29 kg/m^2^.

The case finding process that followed the two recommended algorithms from the EWGSOP1 and EWGSOP2 is described in Figure 2. Pre-sarcopenia was detected in 2.1% (3.1% of males and 0.9% of females) of the participants when following the EWGSOP1-suggested cut-points. Sarcopenia was observed in 0.8% (1.6% of males and 0.0% of females), and 2.5% (4.7% of males and 0.0% of females) were identified to have severe sarcopenia. Using the EWGSOP2-suggested cut-points revealed probable sarcopenia in 5.4% (5.5% of males, 5.3% of females), sarcopenia in 2.9% (5.5% of males, 0.0% of females), and severe sarcopenia in 1.3% (2.4% of males, 0.0% of females) of participants, indicating slightly higher rates in all categories in comparison to the EWGSOP1. The Kosovo-specific cut-points (following the EWGSOP2 algorithm) revealed higher percentages of probable sarcopenia [22.9% (28.3% of males, 16.8% of females)], no cases with sarcopenia [0.0% (0.0% of males, 0.0% of females)], and severe sarcopenia [2.5% (4.7% of males, 0.0% of females)], compared with the EWGSOP2. Taken together, all algorithms confirmed the higher sarcopenic states among the males as compared with the females (EWGSOP1: Chi^2^ = 9.054, *p* = 0.029; EWGSOP2: Chi^2^ = 9.333, *p* = 0.025; EWGSOP2 (Kosovo-specific): Chi^2^ = 10.928, *p* = 0.004).

### 3.3. Impact of EWGSOP2-Derived Sarcopenia States on Health-Related and Socio-Economic Factors

To generate comparable data to already published literature, the EWGSOP2 algorithm and suggested cut-points were used for first analyses. Due to the low number of cases, females were excluded, whereas the comparisons were made between the four groups in males of not sarcopenic (*n* = 110), probable sarcopenic (*n* = 7), sarcopenic (*n* = 7), and severe sarcopenic (*n* = 3). The groups differ in age (F_3,123_ = 5.121, *p* = 0.002, ϖ^2^ = 0.089), height (F_3,123_ = 8.366, *p* < 0.001, ϖ^2^ = 0.164), SMM (F_3,123_ = 6.435, *p* < 0.001, ϖ^2^ = 0.134), ASMM (F_3,123_ = 6.823, *p* < 0.001, ϖ^2^ = 0.086), and ASMI (F_3,123_ = 5.521, *p* = 0.001, ϖ^2^ = 0.031). With respect to the physical performance tests, differences were found for the total performance score (F_3,123_ = 8.554, *p* < 0.001, ϖ^2^ = 0.115), handgrip strength (F_3,123_ = 43.721, *p* < 0.001, ϖ^2^ = 0.513), relative handgrip strength (F_3,123_ = 27.795, *p* < 0.001, ϖ^2^ = 0.482), gait speed (F_3,123_ = 5.926, *p* = 0.001, ϖ^2^ = 0.061), TUG (F_3,123_ = 8.870, *p* < 0.001, ϖ^2^ = 0.056), 30-s arm curl test (F_3,123_ = 4.199, *p* = 0.007, ϖ^2^ = 0.063), 30-s chair stand test (F_3,123_ = 3.020, *p* = 0.032, ϖ^2^ = 0.041), and 6MWT (F_3,123_ = 4.083, *p* = 0.008, ϖ^2^ = 0.059).

Post hoc analyses revealed that the sarcopenic and severe sarcopenic individuals particularly differ from non-sarcopenic ones in most of the variables; the interpretation might be affected by low case numbers. However, handgrip strength (both in absolute and relative measurements) was already lower in males with any of the three sarcopenia conceptual stages compared with the participants without sarcopenia being directly related to the diagnostic algorithm. Most interestingly, the PP score was the only parameter that was able to discriminate between sarcopenia and severe sarcopenia (*p* < 0.05). The number of participants with their marital status described as single (without partner) was lower in the sarcopenic and severe sarcopenic groups (*p* = 0.002), whereas no other lifestyle or socio-economic factor was associated with sarcopenic status (Table 2).

### 3.4. Impact of Kosovan-Specific Cut-Points on Health-Related and Socio-Economic Factors

When comparing the suggested EWGSOP2 cut-points to the Kosovo-derived ones (both using the EWGSOP2 algorithm), it was observed that 179 participants (74.6% of the total population) were classified as non-sarcopenic by both calculation methods, a further 13 subjects (5.4% of total population) with probable sarcopenia, and 2 subjects (0.8% of the total population) with sarcopenia or severe sarcopenia. Furthermore, 46 participants (19.2%) differed in the sarcopenia diagnosis (confirmed by Chi^2^ test: χ^2^ (4) = 84.822, *p* < 0.001). The most striking difference when using the Kosovo-derived cut-points might be the higher number of cases of probable sarcopenia (36 males (28.3%), 19 females (16.8%)). In the next step, we assessed (again only in males) whether the diagnosis was associated with differences in general, anthropometric, physical performance, lifestyle, socio-economic, and health parameters (Table 3).

Sarcopenia was associated with a higher age (F_2,124_ = 8.682, *p* = 0.001, ϖ^2^ = 0.108) but lower height (F_2,124_ = 15.223, *p* < 0.001, ϖ^2^ = 0.183), body mass (F_2,124_ = 11.299, *p* < 0.001, ϖ^2^ = 0.140), BMI (F_2,124_ = 11.693, *p* < 0.001, ϖ^2^ = 0.144), whole body fat mass (F_2,124_ = 3.960, *p* = 0.022, ϖ^2^ = 0.045), whole body fat percentage (F_2,124_ = 3.293, *p* = 0.040, ϖ^2^ = 0.035), skeletal muscle mass (F_2,124_ = 21.463, *p* < 0.001, ϖ^2^ = 0.244), MNA (F_2,124_ = 8.917, *p* < 0.001, ϖ^2^ = 0.111), ASMM (F_2,124_ = 14.101, *p* < 0.001, ϖ^2^ = 0.171), and ASMI (F_2,124_ = 15.823, *p* < 0.001, ϖ^2^ = 0.189). Regarding the physical performance tests, sarcopenic subjects showed a lower total performance score (F_2,124_ = 24.484, *p* < 0.001, ϖ^2^ = 0.270), handgrip strength (F_2,124_ = 114.468, *p* < 0.001, ϖ^2^ = 0.641), gait speed (F_2,124_ = 17.672, *p* < 0.001, ϖ^2^ = 0.208), TUG (F_2,124_ = 10.420, *p* < 0.001, ϖ^2^ = 0.129), 30-s arm curl test (F_2,124_ = 14.790, *p* < 0.001, ϖ^2^ = 0.178), 30-s chair stand test (F_2,124_ = 9.871, *p* < 0.001, ϖ^2^ = 0.123), and 6MWT (F_2,124_ = 8.917, *p* < 0.001, ϖ^2^ = 0.111). Post hoc analyses revealed that handgrip strength was already lower in the probable sarcopenic males, but in contrast to the use of the EWGSOP2 general cut-points, differences were detected between probable and sarcopenic/severe sarcopenic males. Only the skeletal muscle mass was markedly different between all three groups.

Similar to the regular EWGSOP2 analyses, the educational level differed between groups (*p* = 0.035). In addition, participants from the sarcopenic/severe sarcopenic group showed a lower nutritional status (F_2,124_ = 8.917, *p* < 0.001, ϖ^2^ = 0.111) together with a higher percentage of risk for malnourishment (*p* = 0.005) and lower percentages of overweight and obese cases (*p* < 0.001). Interestingly, the self-perceived health or medication intake and socio-economic variables did not differ between the groups (Table 3).

### 3.5. Determinants of Sarcopenic States in Male Kosovan Older Adults (Kosovo-Derived Cut-Points)

To model the relationship between the observed sarcopenia categories (no sarcopenia/probable sarcopenia/severe sarcopenia) and several potential predictors (age, BMI, body fat mass, SMM, PP score, MNA score, and self-perceived financial and health conditions), a multinomial logistic regression was performed using only the data from the male participants. The addition of the predictors to a model that only contained the intercept significantly improved the fit between the model and data [χ^2^ (16) = 51.550, Nagelkerke R^2^ = 0.538, *p* < 0.001]. Age [χ^2^ (2) = 8.604, *p* = 0.014], BMI [χ^2^ (2) = 9.557, *p* = 0.008], body fat mass [χ^2^ (2) = 9.971, *p* = 0.007], and SMM [χ^2^ (2) = 16.043, *p* < 0.001] significantly contributed to the model, whereas PP score [χ^2^ (2) = 4.905, *p* = 0.086], MNA score [χ^2^ (2) = 0.584, *p* = 0.747], financial condition [χ^2^ (2) = 5.431, *p* = 0.066], and health condition [χ^2^ (2) = 5.690, *p* = 0.058] did not.

Table 4 presents the results of the unadjusted and adjusted multinomial logistic regression. Being older and having a lower PP score was associated with a higher risk for being severely sarcopenic in both models (age, adjusted model: RR = 1.03, 95%, CI: 1.01–1.04, *p* = 0.008; PP score, adjusted model: RR = 0.94, 95%, CI: 0.86–1.00, *p* = 0.039). A higher BMI (adjusted model: RR = 1.07, 95%, CI: 1.02–1.10, *p* = 0.009) but lower body fat mass (adjusted model: RR = 0.96, 95% CI: 0.93–0.99, *p* = 0.007) were associated with probable sarcopenia, but not with severe sarcopenia. A lower SMM predicted probable (adjusted model: RR = 0.96, 95% CI: 0.92–0.99, *p* = 0.023) as well as severe sarcopenia (adjusted model: RR = 0.94, 95% CI: 0.90–0.98, *p* = 0.003). A lower reported health condition was associated with severe sarcopenia (adjusted model: RR = 0.34, 95% CI: 0.03–0.99, *p* = 0.045). MNA and financial condition did not contribute to sarcopenia status.

## 4. Discussion

This study aimed to identify the impact of different diagnostic criteria (the initial EWGSOP1 and the revised EWGSOP2) and the recommended and population-specific cut-points on sarcopenia and its conceptual stages in adults aged 60 years and older from a developing European country. Furthermore, it intended to evaluate the impact of socio-economic, health-related, and lifestyle factors on sarcopenia status. The main findings identify the state of sarcopenia within the sample of ageing Kosovans being low, notwithstanding the used algorithms and diagnostic criteria (EWGSOP1, EWGSOP2, and the population-specific cut-points) and the used variable as a key characteristic (SMI vs. muscle strength). Furthermore, even with the lowering of the diagnostic thresholds in the revised EWGSOP2 guideline, no consequences were observed. While rather similar outcomes (0.8%, 2.9%, and 0.0%) were observed in the sarcopenic (EWGSOP1, EWGSOP2, and population-specific cut-points, respectively) and severe sarcopenic (2.5%, 1.3% and 2.5%, respectively) cases, differences were observed in the pre-sarcopenic/probable sarcopenic (2.1%, 5.4% and 22.9%) cases. With respect to this conceptual stage, the EWGSOP2 algorithm (applied in the EWGSOP2 and population-specific cut-points) was able to detect a generally higher percentage, particularly when using the population-specific cut-points. In all cases of pre-sarcopenia/probable sarcopenia (EWGSOP1, EWGSOP2 and population specific), females presented lower percentages (0.9%, 5.3% and 16.8%) in comparison to males (3.1%, 5.5% and 28.3%), and no algorithm could detect sarcopenic or severe sarcopenic females.

It is important to mention that aside from the availability of the various diagnostic cut-points for defining sarcopenia, there is a prevailing lack of general consensus on a robust definition that identifies sarcopenic individuals in different ethnic groups [12]. Furthermore, it has been suggested that until this global consensus is reached, prevalence data should be reported and interpreted within its own context [33]. Upon the publication of the revised EWGSOP2 consensus criteria [3], potentially different outcomes when comparing previous criteria have been investigated [34,35,36,37,38,39]. Reis et al. [34] were among the first to analyze the consequences of applying the new EWGSOP2 guideline instead of the former EWGSOP1 for sarcopenia case finding in older geriatric inpatients and described a substantial mismatch with a significant lowering of the number of men diagnosed with sarcopenia. The discord in prevalence from the lower numbers of the EWGSOP2 have been further explored and reported by other studies [35,36,37,39,40,41]. Besides the several distinguished differences between the two versions (lowering the diagnostic thresholds for isometric handgrip strength and muscle mass, the introduction of new suggested measurement methods for muscle strength, etc.), the shift towards muscle strength from muscle mass (as the major component) probably presents the greatest reason behind the differences.

Inconsistent results exist with respect to the association between sex and sarcopenia [42]; the lower number of (severe) sarcopenic females in this study could be attributed to several intermediate covariates, such as being younger and shorter, similar weight, and consequently a higher BMI. The very high percentage of overweight and obese cases observed in the total participants (43.3% and 41.7%, respectively) together with the higher levels of average BMI, especially among females, could explain the potential impact on the general sarcopenia results. A recent study investigating the potential co-occurrence of several age-related issues including sarcopenia, physical frailty, undernutrition, and obesity found a rather low coexistence between them all, suggesting for a need to assess them individually [43]. These concerning higher overweight and obesity levels could also be one of the determinants for the lower sarcopenic participants in the form that has been previously explained as the “obesity paradox” [44]. This became evident especially after having no cases of sarcopenic obesity. Regarding the prevalence of overweight and obese cases, the rates have been described to vary within European populations, from as low as 53.9% (20.9% male and 16.5% female) and 55.0% (38.7% male and 16.3% female) in Switzerland and Denmark, respectively, to up to 64.0% (40.9% male and 23.1% female) and 67.4% (46.5% male and 20.9% female) in Germany and Spain, respectively [45]. An interesting study from four different countries (Canada, Brazil, Colombia, and Albania) has shown that Albanian men and women aged 65–74 years presented the highest prevalence of overweight and obesity (46.7% and 36.3%) in comparison to their peers [46]. Despite coming from a very specific age group (65–74 years old), Albanian seniors may present the closest similar and comparable population to ours (Kosovo Albanians form the major ethnic group in the country at 95%) [17]; this provides grounded support for our findings. The potential link between the lower number of sarcopenic females (within all conceptual stages) with higher percentages of overweight, obesity, and malnourishment (or being at risk for) might be explained by the significantly lower level of education and financial condition in females, as well as the higher medication intake and number of medications despite no higher level of chronic diseases compared with the males.

In contrast to the females, despite being older, males performed physically better in all physical performance tests except for the 30-s arm curl, where sex-specific weights were applied. The fact that males perform better than their female peers within the same age group in most performance measures has already been described [47], but one must consider that the males were significantly older than the females in the current study. Besides the differences in sex, the high percentages of obesity in females may serve as a further triggering factor, especially due to its inverse association with physical ability [48]. Despite the sex-specific differences in the sarcopenia-related components, overweight and obese instances might also be related and explained through genetic predisposing factors [49], notably through the suggested potential influence that a certain single nucleotide polymorphism (ACTN3 R577X) might exert on knee extensors’ peak torque and BMI, particularly in females [50]. However, not only the gene × gene, but the gene × environment interactions must be further studied to better analyze the interaction.

The strengths of this study lay in the fact that it followed the revised 2019 version of EWGSOP(2); higher age, lower height, SMM, SMI, and ASMM present reliable parameters that significantly change between the different conceptual stages of sarcopenia, lowering as one observes from the non-sarcopenic group to the probable sarcopenic and sarcopenic/severe sarcopenic groups. With respect to the ASMI, it should be noted that it is directly dependent on the subject’s height, which can become less reliable when having a significantly shorter, yet overweight or obese population. Furthermore, the EWGSOP2 are also accompanied by a lowering of physical performance parameters (gait speed, timed up and go, 30-s arm curl, 30-s chair stand, and 6-min walking test) and muscle strength measurements (handgrip strength), which are all significantly related to the occurrence of sarcopenia. Additionally, population-specific cut-off points were able to detect much higher rates of probable sarcopenia than the EWGSOP2-suggested cut-off points. However, this was made possible mainly due to the higher values for the population-specific cut-off points. While taking into consideration the suggestion that the cut-off points used for the muscle mass could affect the reported prevalence rates for sarcopenia [51], the revised EWGSOP2 guidelines’ emphasis should be given with respect to muscle strength.

A recent study [34] raised the point that the importance of the lower number of SMM measurements (in this case using DXA) required in EWGSOP2 guidelines undoubtedly presented an advantage in terms of both availability and cost. While observing the same situation with respect to the Inbody measurements in the community-dwelling older adults while following the EWGSOP2 guidelines, using the suggested key characteristic of strength over muscle mass as a better indicator of the adverse clinical outcomes of mortality and low physical performance [3,52,53] should positively impact the case finding process, particularly in developing lower- and middle-income countries. Therefore, future updates and revisions of the diagnostic approaches (for different populations and population settings) should seriously consider following the EWGSOP2 pathway or attempt to adjust towards using the same logic in order to enhance and offer more robust diagnostic approaches.

Despite performing this study to the best of our scientific knowledge, some limitations emerged that were out of our reach. The first is in regards to the assessment method of muscle mass. Among the several methods available, the usage of BIA in this study instead of DXA, which presents a widely available non-invasive instrument [3,54] and has also been suggested as a reference standard [54], might have overestimated the muscle mass [55]. One study has even suggested the possibility of standing multi-frequency BIA (the same as used in this study) overestimating the fat mass percentage in women with higher BMIs in comparison to both the supine BIA and DXA methods [56]. With respect to the different forms of BIA, good inter-unit precision and no differences between the standing and supine BIA were reported [56,57]. However, BIA as a technique was shown to have an acceptable accuracy [58,59], was recommended for use in research [3], presented a high degree of correlation with the DXA approach [20,60] and magnetic resonance imaging (MRI) [61] in the muscle mass assessment. This, with smaller differences within European populations in comparisons to other methods, all while being affordable, widely available, and portable. Of note, MRI and computerized tomography (CT) techniques, despite being considered the gold standard for a non-invasive assessment of muscle mass, are not commonly used in practice mainly due to their high cost, lack of portability, need for specialized experts [62], and not yet well-defined cut-off points [3]. Another potential limitation of the study was the multivariate regression formula that we used for the DXA estimation of BIA muscle mass measurements, which was initially used for a different population (Koreans) [20]. Lastly, the number of participating subjects might present a limitation for this study, representing less than 1% of the eligible population (within the region) and thus resulting in a margin of error of approximately 6.3% when considered to be a representative population. Nevertheless, besides the inverse relationship of the margin of error with the sample size, the upper threshold for an acceptable marginal error of an estimated sample size has been reported to be not more than 7% at the 97% confidence level [63]. In this context, we believe that the outcomes from this study may provide preliminary data and anticipate potential future directions to follow, according to the current state-of-the-art within the field of the age-related decline of muscle mass and functioning in ageing adults from a developing European country. Furthermore, there is a need to conduct bigger epidemiological studies within these populations in order to further explore and clarify the situation. This must be preceded by recruiting a representative sample of young participants in order to develop and determine more accurate population-specific cut-off points.

## 5. Conclusions

The revised EWGSOP2 algorithm (applied in the EWGSOP2 and population-specific diagnostic criteria) was able to detect generally higher percentages of the pre-sarcopenia/probable sarcopenia conceptual stages, particularly when using the population-specific cut-points. Males presented higher rates and better performance, despite their older age. Higher rates of overweight and obesity could be contributing to the lower number of sarcopenic/severe sarcopenic females, which could also be providing a preventative role in the total population. However, sarcopenia presents a health condition and a growing health-related concern with the ageing of the world population, particularly when taking into consideration the serious health implications that it unveils. To date, prevention and early detection present the strongest instruments within our reach to tackle this issue. Additionally, the introduction and promotion of sarcopenia together with its implications is mandatory for healthcare providers and others directly involved in dealing with older persons in developing countries. Additionally, population-specific diagnostic cut-off points should be developed for different populations to create a very accurate and versatile instrument for screening and diagnostic purposes. Further studies are required to examine the impact of weight on sarcopenia in the study’s population.

The higher levels of overweight and obesity that were found in the study participants (Kosovan older adults) should be taken as a serious concern of public health, keeping in mind that the obesity epidemic in industrialized countries displays a very critical risk factor for chronic diseases and has a heavy impact on health, quality of life, and life expectancy [64]. Considering these facts, the very high level of overweight and obesity in our study population could be one of the direct factors triggering the main cause of death in Kosovo (cardiovascular diseases) [65], consequently resulting in the lower expected life expectancy at birth in this country.

## Figures and Tables

**Table 1:** Anthropometric, physical performance, lifestyle, and socio-demographic characteristics.

	Total (*n* = 240)	Female (*n* = 113)	Male (*n* = 127)	*p* Value
Sex (%)	100	47.1	52.9	
Age (years)	70.3 ± 5.8	68.4 ± 5.3	72.1 ± 5.7	<0.001
Height (m)	1.64 ± 0.09	1.57 ± 0.06	1.70 ± 0.07	<0.001
Body mass (kg)	79.9 ± 12.7	79.3 ± 11.7	80.4 ± 13.6	0.504
BMI (kg/m^2^)	29.7 ± 4.7	32.0 ± 4.3	27.7 ± 4.2	<0.001
Whole body fat mass (kg)	29.6 ± 11.1	35.3 ± 8.9	24.4 ± 10.3	<0.001
Whole body fat percentage (%)	36.4 ± 10.6	44.1 ± 6.5	29.6 ± 8.6	<0.001
Skeletal muscle mass (kg)	26.9 ± 5.4	23.3 ± 3.2	30.2 ± 5.0	<0.001
SMI (kg/m^2^)	9.9 ± 1.2	9.4 ± 0.9	10.3 ± 1.3	<0.001
Appendicular skeletal muscle mass (kg)	19.4 ± 2.9	18.2 ± 2.2	20.5 ± 3.1	<0.001
ASMI (kg/m^2^)	7.2 ± 0.8	7.3 ± 0.7	7.0 ± 0.8	0.003
Hand grip strength (kg)	30.1 ± 8.8	24.1 ± 5.1	35.4 ± 8.0	<0.001
Gait speed (m/s)	1.08 ± 0.21	1.01 ± 0.19	1.14 ± 0.22	<0.001
Timed up and go test (s)	7.12 ± 1.98	7.50 ± 2.20	6.78 ± 1.70	0.005
30-s arm curl test (repetitions)	14 ± 3	14 ± 3	15 ± 3	0.100
30-s chair stand test (repetitions)	11 ± 3	11 ± 3	12 ± 3	0.004
6-min walking test (m)	420 ± 139	381 ± 127	455 ± 140	<0.001
Physical performance Score (-)	−1.26 ± 1.86	−1.78 ± 1.81	0.8 ± 1.79	<0.001
Mini nutritional status (-)	25 ± 3	24 ± 3	25 ± 2	<0.001
Malnourished (yes/risk/no, *n* (%))	4/64/172 (1.7/26.6/71.7)	4/41/68 (3.5/36.3/60.2)	0/23/104 (0.0/18.1/81.9)	<0.001
BMI categories (underweight/normal weight/overweight/obese, *n* (%))	2/34/104/100 (0.8/14.2/43.3/41.7)	0/7/35/71 (0.0/6.2/31.0/62.8)	2/27/69/29 (1.6/21.3/54.3/22.8)	<0.001
Smoking status (smoker/quit smoking/non-smoker, *n* (%))	53/24/163 (22.1/10/67.9)	20/4/89 (17.7/3.5/78.8)	33/20/74 (26/15.7/58.3)	0.001
Self-perceived health condition (good/not good, *n* (%))	103/137 (42.9/57.1)	50/63 (44.2/55.8)	53/74 (41.7/58.3)	0.694
Self-declared chronic disease (yes/no, *n* (%))	183/57 (76.2/23.8)	90/23 (79.6/20.4)	93/34 (73.2/26.8)	0.244
Intake of medication (yes/no, *n* (%))	186/54 (77.5/22.5)	95/18 (84.1/15.9)	91/36 (71.6/28.4)	0.021
Number of medications (*n* (%))	2.3 ± 1.9	2.8 ± 1.9	1.9 ± 1.7	<0.001
Education (no formal/1–8 years/>8 years, *n* (%))	10/95/135 (4.2/39.6/56.2)	7/62/44 (6.2/54.9/38.9)	3/33/91 (2.4/26/71.6)	<0.001
Marital status (single/partnership or married/widowed, *n* (%))	7/166/67 (2.9/69.2/27.9)	7/66/40 (6.2/58.4/35.4)	0/100/27 (0/78.7/21.3)	0.003
Financial condition (enough to cover the month/not enough, *n* (%))	166/74 (69.2/30.8)	70/43 (61.9/38.1)	96/31 (75.6/24.4)	0.022

Data are expressed as the means ± standard deviation or as absolute numbers; Abbreviations: BMI, body mass index; SMI, skeletal muscle index; ASMI, appendicular skeletal muscle index; m, meters; kg, kilograms; s, seconds. An independent *t*-test was used to determine the differences between females and males in the continuous variables and a chi-square test was used in the categorical variables; *p* < 0.05 was considered to be statistically significant.

**Table 2:** Differences between the non-sarcopenic, probable sarcopenic, sarcopenic, and severe sarcopenic male subjects using the EWGSOP2 criteria.

	No Sarcopenia (*n* = 110)	Probable Sarcopenia (*n* = 7)	Sarcopenia (*n* = 7)	Severe Sarcopenia (*n* = 3)	*p* Value
Age(years)	71.4 ± 5.2 ^a^	74.5 ± 8.1 ^a,b^	79.2 ± 6.7 ^b^	73.9 ± 0.8 ^a,b^	0.002
Height (m)	1.71 ± 0.06 ^a^	1.65 ± 0.07 ^b^	1.61 ± 0.10 ^b^	1.66 ± 0.08 ^a,b^	<0.001
Body mass (kg)	81.2 ± 14.2	78.3 ± 5.3	74.5 ± 7.6	69.5 ± 3.3	0.280
BMI (kg/m^2^)	27.6 ± 4.3	28.9 ± 1.7	28.8 ± 4.5	25.4 ± 2.7	0.579
Whole body fat mass (kg)	24.7 ± 10.7	22.5 ± 4.7	23.5 ± 11.2	22.7 ± 4.0	0.933
Whole body fat percentage (%)	29.4 ± 8.4	29.0 ± 6.7	31.7 ± 14.6	32.6 ± 4.1	0.830
Skeletal muscle mass (kg)	30.9 ± 4.5 ^a^	26.9 ± 6.0 ^a,b^	24.5 ± 7.0 ^b^	25.3 ± 0.7 ^a,b^	<0.001
SMI (kg/m^2^)	10.5 ± 1.2 ^a^	9.9 ± 1.7 ^a^	9.3 ± 2.1 ^a^	9.2 ± 0.9 ^a^	0.037
Appendicular skeletal muscle mass (kg)	23.7 ± 3.5 ^a^	23.2 ± 3.1 ^a^	18.3 ± 3.4 ^b^	19.6 ± 0.7 ^a,b^	<0.001
ASMI (kg/m^2^)	8.0 ± 0.9 ^a^	8.6 ± 0.9 ^a^	7.0 ± 0.6 ^b^	7.2 ± 0.5 ^a,b^	0.001
PP score (-)	1.06 ± 2.56 ^a^	−0.31 ± 2.08 ^a^	−0.86 ± 1.87 ^a^	−5.65 ± 0.66 ^b^	<0.001
Handgrip strength (kg)	37.7 ± 5.8 ^a^	22.6 ± 2.7 ^b^	19.8 ± 4.3 ^b^	19.8 ± 7.8 ^b^	<0.001
Relative handgrip strength (kg/kg)	0.47 ± 0.08 ^a^	0.29 ± 0.03 ^b^	0.27 ± 0.06 ^b^	0.28 ± 0.10 ^b^	<0.001
Gait speed (m/s)	1.16 ± 0.22 ^a^	1.08 ± 0.14 ^a^	1.04 ± 0.14 ^a,b^	0.68 ± 0.14 ^b^	0.001
Timed up and go test (s)	6.64 ± 1.52 ^a^	6.63 ± 0.91 ^a^	7.13 ± 2.37 ^a^	11.30 ± 2.01 ^b^	<0.001
30-s arm curl test (repetitions)	15 ± 3 ^a^	13 ± 3 ^a,b^	13 ± 3 ^a,b^	10 ± 2 ^b^	0.007
30-s chair stand test (repetitions)	12 ± 3 ^a^	12 ± 3 ^a^	10 ± 2 ^a,b^	8 ± 2 ^b^	0.032
6-min walking test (m)	470 ± 139 ^a^	369 ± 82 ^a,b^	384 ± 121 ^a,b^	261 ± 92 ^b^	0.008
Mini nutritional status (-)	25 ± 2	25 ± 3	26 ± 2	22 ± 3	0.059
Malnourished (yes/risk/no, *n* (%))	19/91 (17.3/82.7)	1/6 (14.3/85.7)	0/1/6 (0/14.3/85.7)	0/2/1 (0/66.7/33.3)	0.175
BMI categories (underweight/normal weight/overweight/obese, *n* (%))	2/24/60/24 (1.8/21.8/54.5/21.8)	0/0/5/2 (0.0/0.0/71.4/28.6)	0/2/2/3 (0/28.6/28.6/42.9)	0/1/2/0 (0/33.3/66.7/0)	0.781
Smoking status (smoker/quit smoking/non-smoker, *n* (%))	31/19/60 (28.2/17.4/54.5)	0/0/7 (0.0/0.0/100.0)	2/0/5 (28.6/0/71.4)	0/1/2 (0/33.3/66.7)	0.212
Self-perceived health condition (good/not good, *n* (%))	48/62 (43.6/56.4)	3/4 (42.9/57.1)	2/5 (28.6/71.4)	0/3 (0/100)	0.421
Self-declared chronic disease (yes/no, *n* (%))	80/30 (72.7/27.3)	4/3 (57.1/42.9)	6/1 (85.7/14.3)	3/0 (100/0)	0.459
Intake of medication (yes/no, *n* (%))	19/91 (17.3/82.7)	3/4 (42.9/57.1)	1/6 (14.3/85.7)	2/1 (66.7/33.3)	0.069
Number of medications (-)	1.9 ± 1.7	1.9 ± 1.8	1.9 ± 1.8	3.0 ± 1.7	0.741
Education (no formal/1–8 years/>8 years, *n* (%))	2/25/83 (1.8/22.7/75.5)	0/2/5 (0.0/28.6/71.4)	1/4/2 (14.3/57.1/28.6)	0/2/1 (0/66.7/33.3)	0.057
Marital status (single/partnership or married/widowed, *n* (%))	0/88/22 (0/80.0/20.0)	0/6/1 (0/85.7/14.3)	0/5/2 (0/71.4/28.6)	0/1/2 (0/33.3/66.7)	0.002
Financial condition (enough to cover the month/not enough, *n* (%))	85/25 (77.3/22.7)	4/3 (57.1/42.9)	6/1 (85.7/14.3)	1/2 (33.3/66.7)	0.191

Data shown as the means ± standard deviations or the absolute and relative frequencies. Differences were analyzed by a one-factorial ANOVA test followed by Tukey HSD post hoc tests. Different superscript letters (“^a^” and “^b^”) indicate the statistically significant differences. Differences between the categorical data were analyzed by chi-square tests. *p* < 0.05 was considered to be statistically significant. Abbreviations: ASMI (appendicular skeletal muscle index), BMI (body mass index), PP (physical performance), and SMI (skeletal muscle index).

**Table 3:** Differences between the non-sarcopenic, probable sarcopenic, and sarcopenic male subjects using the Kosovo-specific cut-points following the EWGSOP2 algorithm.

	No Sarcopenia (*n* = 85)	Probable Sarcopenia (*n* = 36)	Severe Sarcopenia (*n* = 6)	*p* Value
Age (years)	70.7 ± 4.5 ^a^	75.0 ± 6.4 ^a^	74.2 ± 10.0 ^a^	<0.001
Height (m)	1.72 ± 5.51 ^a^	1.66 ± 7.51 ^a,b^	1.70 ± 4.15 ^b^	<0.001
Body mass (kg)	83.0 ± 14.0 ^a^	78.0 ± 9.3 ^a^	58.7 ± 8.0 ^b^	<0.001
BMI (kg/m^2^)	27.9 ± 4.1 ^a^	28.5 ± 3.6 ^a^	20.3 ± 2.1 ^b^	<0.001
Whole body fat mass (kg)	25.2 ± 10.6 ^a^	24.5 ± 9.1 ^a^	13.2 ± 7.5 ^b^	0.022
Whole body fat percentage (%)	29.5 ± 7.8 ^a^	31.1 ± 9.6 ^a^	21.5 ± 10.7 ^b^	0.040
Skeletal muscle mass (kg)	31.7 ± 3.9 ^a^	27.7 ± 5.2 ^b^	22.3 ± 4.6 ^c^	<0.001
SMI (kg/m^2^)	10.7 ± 1.0 ^a^	10.0 ± 1.4 ^a^	7.7 ± 1.4 ^b^	<0.001
Appendicular skeletal muscle mass (kg)	21.2 ± 2.9 ^a^	19.5 ± 2.6 ^a^	15.6 ± 1.3 ^b^	<0.001
ASMI (kg/m^2^)	8.2 ± 0.8 ^a^	7.9 ± 0.9 ^a^	6.3 ± 0.5 ^b^	<0.001
PP score (-)	1.72 ± 2.29 ^a^	−1.15 ± 2.27 ^b^	−2.19 ± 2.78 ^b^	<0.001
Handgrip strength (kg)	39.9 ± 4.5 ^a^	26.7 ± 4.7 ^b^	24.0 ± 8.7 ^b^	<0.001
Relative handgrip strength (kg/kg)	0.49 ± 0.07 ^a^	0.35 ± 0.07 ^a^	0.43 ± 0.20 ^b^	<0.001
Gait speed(m/s)	1.21 ± 0.20 ^a^	1.00 ± 0.19 ^b^	0.95 ± 0.13 ^b^	<0.001
Timed up and go test (s)	6.37 ± 1.39 ^a^	7.40 ± 1.90 ^a,b^	8.77 ± 2.10 ^b^	<0.001
30-s arm curl test (repetitions)	16 ± 3 ^a^	12 ± 3 ^b^	12 ± 3 ^b^	<0.001
30-s chair stand test (repetitions)	12 ± 3 ^a^	10 ± 2 ^a,b^	10 ± 3 ^b^	<0.001
6-min walking test (m)	489 ± 138 ^a^	390 ± 113 ^a,b^	355 ± 149 ^b^	<0.001
Mini nutritional status (-)	26 ± 2 ^a^	25 ± 2 ^a^	22 ± 3 ^b^	<0.001
Malnourished (yes/risk/no, *n* (%))	12/73 (14.1/85.9)	7/29 (19.4/80.6)	4/2 (66.7/33.3)	0.005
BMI categories (underweight/normal/overweight/obese, *n* (%))	0/17/50/18 (0.0/20.0/58.8/21.2)	0/6/19/11 (0.0/16.7/52.8/30.6)	2/4/0/0 (33.3/66.7/0.0/0.0)	<0.001
Smoking status (smoker/quit smoking/non-smoker, *n* (%))	24/14/47 (28.2/16.5/55.3)	5/5/26 (13.9/13.9/72.2)	4/1/1 (66.7/16.7/16.7)	0.055
Self-perceived health condition [good/not good, *n* (%)]	38/47 (44.7/55.3)	11/25 (30.6/69.4)	4/2 (66.7/33.3)	0.158
Self-declared chronic disease (yes/no, *n* (%))	62/23 (72.9/27.1)	27/9 (75/25)	4/2 (66.7/33.3)	0.908
Intake of medication (yes/no, *n* (%))	63/22 (74.1/25.9)	24/12 (66.7/33.3)	4/2 (66.7/33.3)	0.681
Number of medications (-)	1.9 ± 1.7	2.0 ± 1.7	1.0 ± 1.7	0.406
Education (no formal/1–8 years/>8 years, *n* (%))	0/20/65 (0.0/23.5/76.5)	2/11/23 (5.6/30.6/63.9)	1/2/3 (16.7/33.3/50.0)	0.035
Marital status (single/partnership or married/widowed, *n* (%))	0/70/15 (0/82.4/17.6)	0/25/11 (0/69.4/30.6)	0/5/1 (0/83.3/16.7)	0.273
Financial condition (enough to cover the month/not enough, *n* (%))	65/20 (76.5/23.5)	28/8 (77.8/22.2)	3/3 (50.0/50.0)	0.323

Data shown as the means ± standard deviations or the absolute and relative frequencies. Differences were analyzed by a one-factorial ANOVA test followed by Tukey HSD post hoc tests. Different superscript letters (“^a^”, “^b^” and “^c^”) indicate the statistically significant differences between groups. Differences between the categorical data were analyzed by chi-square tests. *p* < 0.05 was considered to be statistically significant. Abbreviations: ASMI (appendicular skeletal muscle index), BMI (body mass index), PP (physical performance), and SMI (skeletal muscle index).

**Table 4:** Multinomial logistic regression predicting sarcopenia status.

	Unadjusted Model		Adjusted Model			
	Probable Sarcopenia	Severe Sarcopenia	Probable Sarcopenia	Severe Sarcopenia	Probable Sarcopenia	Severe Sarcopenia
	OR (95% CI)	OR (95% CI)	OR (95% CI)	OR (95% CI)	RR (95% CI)	RR (95% CI)
Age (years)	1.099 (0.971–1.244)	1.172 (1.058–1.299) **	1.034 (0.890–1.200)	1.227 (1.054–1.427) **	1.004 (0.984–1.023)	1.025 (1.007–1.042) **
BMI (kg/m^2^)	1.067 (0.908–1.254)	1.012 (0.868–1.179)	1.928 (1.182–3.147) **	1.351 (0.896–2.039)	1.069 (1.021–1.100) **	1.036 (0.985–1.073)
Body fat mass (kg)	0.978 (0.900–1.062)	0.986 (0.922–1.054)	0.784 (0.658–0.935) **	0.864 (0.725–1.029)	0.965 (0.935–0.991) **	0.979 (0.952–1.004)
SMM (kg)	0.836 (0.711–0.984) *	0.775 (0.667–0.900) ***	0.770 (0.615–0.965) *	0.693 (0.546–0.880) **	0.962 (0.923–0.995) *	0.944 (0.900–0.982) **
PP score (-)	0.814 (0.606–1.094)	0.611 (0.461–0.811) ***	0.959 (0.621–1.481)	0.659 (0.443–0.980) *	0.994 (0.924–1.045)	0.935 (0.856–0.997) *
MNA score (-)	0.978 (0.716–1.336)	0.893 (0.695–1.147)	0.986 (0.613–1.586)	1.210 (0.717–2.045)	0.998 (0.922–1.052)	1.024 (0.950–1.073)
Financial condition ^a^						
Not enough to cover the month	0.392 (0.082–1.870)	0.686 (0.165–2.851)	0.106 (0.010–1.125)	0.156 (0.012–1.949)	0.469 (0.070–1.015)	0.580 (0.086–1.070)
Health condition ^b^						
Not good	0.969 (0.207–4.535)	0.323 (0.066–1.591)	0.692 (0.087–5.486)	0.063 (0.004–0.941) *	0.944 (0.417–1.123)	0.335 (0.031–0.992) *

No sarcopenia was chosen as the reference group for the outcome. ^a^ Reference category is “enough to cover the month”; ^b^ Reference category is “good”. In the unadjusted model, each variable was independently tested as a predictor of sarcopenia status. In the adjusted model, all the variables were tested in the same model, controlling the effect of each other. The RR was estimated from the OR using the conversion formula from Zhang and Yu [32]. * *p* < 0.05, ** *p* < 0.001, *** *p* < 0.001. OR, odds ratio; RR, relative risk; CI, confidence interval; BMI, body mass index; SMM, skeletal muscle mass; PP, physical performance, MNA, Mini Nutritional Assessment.

## Data Availability

The data presented in this study are available on request from the corresponding author.
